# Recurrent desmoid tumor of the mediastinum: A case report

**DOI:** 10.3892/ol.2014.2431

**Published:** 2014-08-08

**Authors:** YUXIN XIE, KEQI XIE, QIHENG GOU, JINLAN HE, LAN ZHONG, YONGSHENG WANG

**Affiliations:** 1 Department of Thoracic Oncology, Cancer Center, State Key Laboratory of Biotherapy, West China Hospital, Sichuan University, Chengdu, Sichuan 610041, P.R. China; 2 Department of Anesthesiology, Mianyang Central Hospital, Mianyang, Sichuan 621000, P.R. China; 3 Department of Pathology, West China Hospital, Sichuan University, Chengdu, Sichuan 610041, P.R. China

**Keywords:** desmoid tumor, recurrent tumor, mediastinum, computed tomography

## Abstract

Desmoid tumors (DTs) are rare, benign soft-tissue tumors that have the potential for local invasion, but not for metastasis. The tumors are commonly characterized by a palpable mass, but present a variable and unpredictable clinical course. The current study presents the case of a giant mediastinal DT exhibiting lung involvement. A 50-year-old female was referred to the West China Hospital (Chengdu, Sichuan, China) due to a recurrent DT that was identified one year following radical surgery. The patient subsequently received radiation therapy. The DT arose from the mediastinum, unlike the usual presentation, and recurrence presented as extensive invasion into the lung tissue, almost being misdiagnosed as lung cancer with brain metastasis. Tumor recurrence was diagnosed through contrast-enhanced computed tomography and histological examination of the tumor. A routine follow-up revealed no further tumor progression at 9 months post-admission. Taking into account the unpredictable treatment complications, recurrent DTs can be managed simply and efficiently. A ‘wait-and-see’ policy could be a viable therapeutic option for this disease.

## Introduction

Desmoid tumors (DTs) are histologically benign monoclonal myofibroblastic neoplasms. However, these tumors tend to be locally invasive and infiltrate into the surrounding soft tissue, but are lacking in metastatic potential (1).

Numerous studies have demonstrated that DTs account for just 0.03% of all neoplasms and ~3% of all soft-tissue tumors (2–5). The majority of cases occur between the ages of 15 and 60 years old, with a peak incidence between 25 and 35 years old (6). The exact etiology of DTs is complicated and remains unknown, however, genetic abnormalities, such as familial adenomatous polyposis and Gardner’s syndrome, and endocrine and physical factors play a role in the pathogenesis of DTs (7,8). These tumors commonly occur in the abdominal wall, followed by intra- or extra-abdominal occurrences, including occurrences in the chest wall and shoulder (9,10). Nevertheless, there have been few reported cases of DTs originating from the lung. The present study reports the computed tomography (CT) and pathological diagnosis findings of a recurrent DT arising from the mediastinum in a 50-year-old female following treatment with surgery and postoperative radiation. Patient provided written informed consent.

## Case report

**Figure 1:**
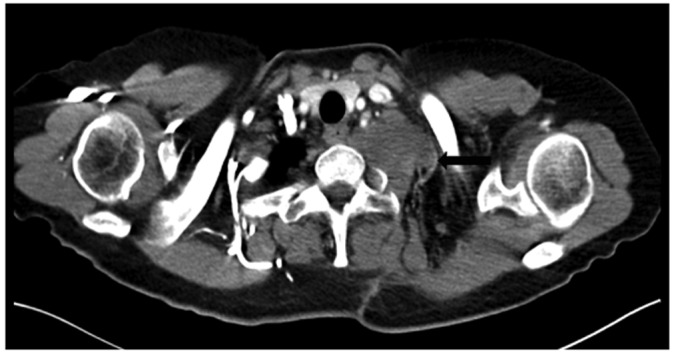
Computed tomography scan of the recurrent desmoid tumour (black arrow) wrapping around the left subclavian artery and causing erosion of the adjoining ribs in November 2012.

**Figure 2:**
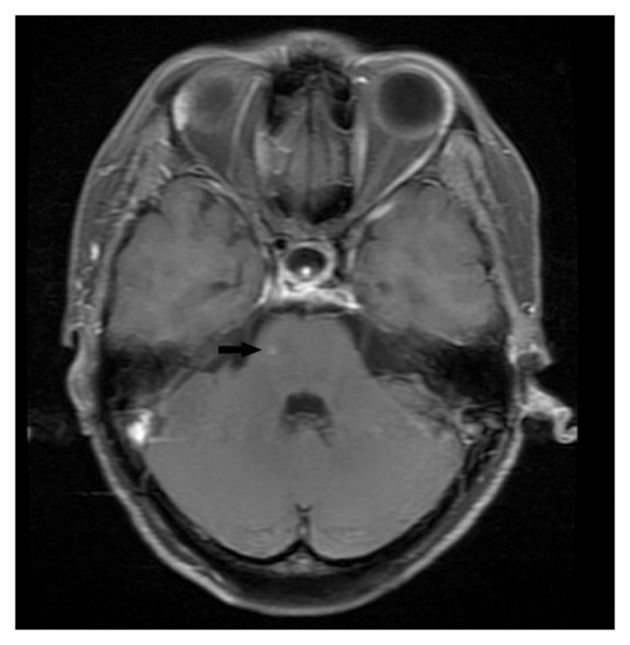
Brain magnetic resonance imaging of a nodule (black arrow) measuring 0.3 cm in the right side of the pons in November 2012.

**Figure 3:**
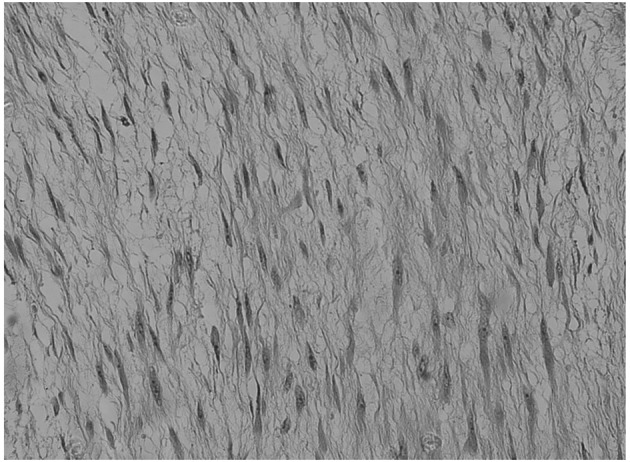
Histopathology of the circumscribed tumor composed of spindle cells with bland nuclei and extracellular collagen (hematoxylin and eosin stain; magnification, ×400).

A 50-year-old female presented with a dull pain in the left scapular region and a decreased range of motion of the left upper limb that had been present for five years. The patient was treated at a local hospital in June 2009. A computed tomography (CT) scan revealed a homogenous soft-tissue density mass lesion in the superior lobe of the left lung. Surgery was performed within the left side of the upper mediastinum and the tumor was completely removed. The longest diameter of the mass was ~6.1 cm. Surgical biopsies of the mass revealed a fibroma. However, in the year following surgery, the pain in the left scapular region occurred again and slowly progressed. Tumor recurrence was diagnosed through a CT scan and histological examination of the tumor. The left chest wall of the patient was treated with radiation therapy (64 Gy for 32 fractions). However, no notable improvement was found. The patient was referred to the West China Hospital (Chengdu, Sichuan, China) for a recurrent tumor of the mediastinum in November 2012. A CT scan revealed that the left thoracic wall had collapsed and there were two soft-tissue masses, located in the left pulmonary apex and the costal pleura below the left plumonary apex, respectively. The maximum cross-section was ~3.3×2.6 cm. The first tumor was wrapped around the left subclavian artery and caused erosion of the adjoining ribs (Fig. 1). The number of inflammatory nodules increased. Furthermore, brain magnetic resonance imaging (MRI) showed a nodule measuring 0.3 cm in the right side of the pons (Fig. 2). A detailed general examination showed no other metastatic or primary lesions. Histopathology revealed a circumscribed tumor composed of spindle cells with bland nuclei and extracellular collagen in the stroma (Fig. 3). The lesion was typical of a fibromatosis (DT). A CT-guided puncture biopsy of the mediastinal mass failed to provide a further pathological diagnosis. The nature of the specific lesions in the pons was also not clear. Since the recurrent DTs were extensively invasive, radical surgery may have been difficult or even impossible. Considering that the quality of life of the patient was not affected and to avoid the potential complications of surgery or other treatments, the patient was treated with a ‘wait-and-see’ policy. A 9-month clinical follow-up was carried out, whereby CT scan (August 2013) revealed that the left thoracic wall had collapsed and the presence of two soft-tissue masses, located in the left pulmonary apex and the costal pleura below the left pulmonary apex, respectively. In comparison to the previous CT scan (November 2012), no changes in the masses were identified. In addition, no changes in the size of the mediastinal lymph nodes were identified. Brain MRI (August 2013) revealed a nodule measuring 0.3 cm in the right side of the pons. Following the CT and MRI scans, the patient was determined to be in a stable condition.

## Discussion

In the present case, the DT originated from the mediastinum, which is a rare location, and the recurrence occurred one year after the initial surgery. However, the range of the recurrent mass was wide and infiltrated up into the neck and down into the lung. When purely analyzed by imaging, the tumor could be misdiagnosed as a malignant tumor arising from the lung. The most puzzling finding is that MRI revealed a nodule that could be considered as brain metastasis in the right side of the pons.

Thus, the definitive diagnosis, classification and differential diagnosis of DTs require a histopathological examination (1,11). However, fine-needle aspiration may not be useful due to the hypocellularity of this tumor (12). In the present case, one attempt was made to obtain a pathological diagnosis of the patient by CT-guide percutaneous lung puncture biopsy during the follow-up period, however, this failed.

Complete surgical excision with wide tumor-free margins is the current treatment for primary and recurrent DTs (11). However, a ‘wait-and-see’ policy (13,14) was adopted as the therapeutic option for the patient in the present study. The patient exhibited a recurrent and widely invasive DT, and the left thoracic wall had collapsed due to the primary wide radical resection, therefore, a complete excision through reoperation would have been difficult to achieve. Considering the unpredictable treatment complications and the increased risk of mortality, only a long-term follow-up was carried out. Furthermore, a previous study has reported that reoperations are associated with a high risk of local recurrence (15). An individualized and comprehensive evaluation was required for the treatment of the patient in the present study, and in fact, the recurrent DT and the nodule in the right side of the pons were stable during the 9-month follow-up and showed no brain metastasis.

Age, tumor location and margin status are all factors associated with recurrence (16). If the DT becomes progressive, a multimodal concept should be followed and another treatment should be used singly or in combination, including chemotherapy (17), radiation therapy (18), hormonal therapy and targeted therapy, such as the use of tyrosine kinase inhibitors (19).

In conclusion, the early detection of DTs and the use of a complete surgical resection play an important role in the prognosis. Long-term follow-up is an indispensable guide to future treatment.

## Figures and Tables

**Figure 4:**
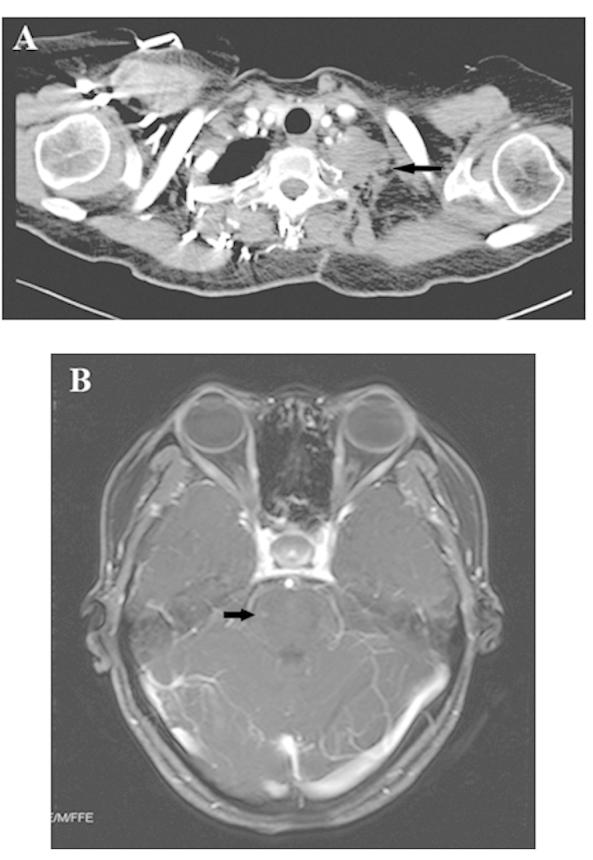
Computed tomography and brain magnetic resonance imaging of (A) the stable desmoid tumor in the lung (black arrow) and (B) the stable nodule (black arrow) in the right of the pons, respectively, at the 9-month follow-up in August 2013.
